# The Waggle Dance as an Intended Flight: A Cognitive Perspective

**DOI:** 10.3390/insects10120424

**Published:** 2019-11-25

**Authors:** Randolf Menzel

**Affiliations:** Institut für Neurobiologie, Freie Universität Berlin, Königin Luisestr. 1-3, 14195 Berlin, Germany; menzel@neurobiologie.fu-berlin.de; Tel.: +49-308-385-6441

**Keywords:** navigation, social communication, symbolic communication, intentions, *Apis mellifera*

## Abstract

The notion of the waggle dance simulating a flight towards a goal in a walking pattern has been proposed in the context of evolutionary considerations. Behavioral components, like its arousing effect on the social community, the attention of hive mates induced by this behavior, the direction of the waggle run relative to the sun azimuth or to gravity, as well as the number of waggles per run, have been tentatively related to peculiar behavioral patterns in both solitary and social insect species and are thought to reflect phylogenetic pre-adaptations. Here, I ask whether these thoughts can be substantiated from a functional perspective. Communication in the waggle dance is a group phenomenon involving the dancer and the followers that perform partially overlapping movements encoding and decoding the message respectively. It is thus assumed that the dancer and follower perform close cognitive processes. This provides us with access to these cognitive processes during dance communication because the follower can be tested in its flight performance when it becomes a recruit. I argue that the dance message and the landscape experience are processed in the same navigational memory, allowing the bee to fly novel direct routes, a property understood as an indication of a cognitive map.

## 1. Introduction

Karl von Frisch discovered that European honeybees perform a waggle dance that communicates at least to the experimenter, the direct flight path to the indicated goal (a feeding site or a new nesting site) [1]). It was difficult for Karl von Frisch and his co-workers to prove that the dance message was also received and applied by the bees following the dance—the recruits—since the necessary methods were not available at that time to fully exclude any guidance by odors. Meanwhile the case is settled, and it is well documented that indeed the recruits perform their outbound search flights according to the dance message [2]. Thus, the waggle dance encodes the direct flight path in a symbolically communicated vector. The flight direction in relation to the current azimuth of the sun is given in the angle of the waggle phase relative to gravity on the vertical comb in the dark hive. The distance from the hive to the indicated location is measured visually [3] and encoded in the number of waggle movements (and possibly other associated parameters, such as waggle time, the length of the waggle run, or the time taken to complete a rotation). Thus, the dance and the corresponding outbound flights by both the dancer and the recruits can be viewed as being defined by a self-centered, egocentric reference, namely the vector or rhumb line from the hive to the goal.

It is remarkable that this kind of symbolic communication of a location is not known to exist in any non-human animal. In our context, it is interesting to ask what is being communicated: Is it the outbound flight vector (as I have cautiously called it above) or rather the location, thus a place defined by allocentric references? In the first case, the recruit would use only the information about the direct flight path from the hive to the communicated goal and follow this instruction without referring to her own memory of the landscape. In the second case, the recruit would interpret the communicated vector as being embedded in her memory and recall the flight along the reported vector and its end point from its memory. In that case, the recruit would steer towards a location along a flight path in a memorized landscape. Memorized means in this context that the dancer as well as the recruit may have expectations about what will be experienced on the outbound flight. Such a cognitive performance based on expectation might reflect an intention (see below).

The purely egocentric interpretation of the waggle dance can be challenged by multiple observations. For example, dancers that have never experienced the outbound flight path, but only the inbound perform perfect dances [4]. Nest scouts that had discovered a superior nest side inhibit another scout’s efforts to continue advertising for an inferior nest site [5] because they have not experienced the vector they reported in their dances. Recruits that have experience with the indicated goal are more likely to fly there even after following a lower number of waggle runs than experienced recruits [6]. A food or nest site is characterized not only by its spatial location but also by a series of characteristics, which are stored in the navigation memory. These include, for instance, the spatial reference to landmarks (its allocentric reference), the route to the place, and the fact that the recruit might already have experience, or indeed no experience, of the place that she has (or does not have) visited before. If the attending bee has gained such experience of the indicated location (e.g., how rich the feeding site is, what odor it has, how the flower is to be manipulated, whether nectar or pollen is to be collected, etc.) then she may make her decision about whether she will follow the dance information dependent on that experience [6]. In such a case, the recruit would have certain expectations not only regarding the place, but also regarding its properties and the landscape characteristics she can expect to encounter en route to, and around, that location. From these contrasting formulations it becomes clear that completely different assumptions can be made about the cognitive processes involved in an egocentric vs. allocentric communication process, decision-making, and subsequent navigation.

It is difficult to avoid an assumption of intentionality involved in this communication process because practically all the signals attached to the performance of the flight are not accessible during the communication process inside the dark hive, and they need to be interpreted on the basis of the navigation memory of the receiving bee. Taking the perspective of the dancer, one could argue that the waggle run mirrors its next outbound flight, a performance that will follow when she leaves the hive. In this sense, the dance could be understood as an intended flight. Similarly, the recruit would learn a simulated outbound flight by repeating the movements of the dancer, store it as an intended flight, and then apply it.

## 2. Results and Discussion

### 2.1. Functional Components and Phylogenetic Routes of the Waggle Dance

The idea of the waggle dance as an intended flight has been put forward in the context of potential phylogenetic pre-adaptations in other insect species ([1] Chapter 11, [7] p. 266 ff), and comparisons of dance behavior in other *Apis s*pecies. The behaviors of non-Hymenoptera can be considered as phylogenetic pre-adaptations only in a very general sense, meaning that particular movement patterns exist across insect species. The most basic component is thought to be food-induced arousal, seen, for example, in the circling “dances” of the fly *Phormia regina* [8,9]. Hungry flies run around a droplet of sugar water and continue performing such “dances” even when the sugar droplet is removed. When the surface is tilted from horizontal to vertical, they transfer the elongated runs they do, relative to light on the horizontal surface, to the upward direction on the vertical surface. When other flies are close, the “dancer” may regurgitate some of the sugar syrup, leading to similar circling movements in the other flies. Dance-like movements are known from multiple insect species in the context of courtship. For example, male flies of the genus *Lispe* perform dance-like motion patterns during courtship [10], and males of the hoverfly *Syritta pipiens* track the movements of conspecifics [11], a skill that seemingly serves copulatory functions.

A flight-dependent form of arousal was observed in many species of Saturniid moths [12]. The side-to-side oscillations of the whole body follow immediately after landing and appear to last longer after more extended flights. More complex forms of arousal movements are seen in social hymenopterans (bumble bees [13], stingless bees [14]). These agitating runs are often combined with vibratory signals that are likely to attract attention from colony members [15]. Information about the food source may be transferred in the colony because the odors on the body of agitating bumble bees will be perceived by other bees [16]. In another bumble bee study, no recruitment to unscented food sources was observed [17]. The situation is different in stingless bees like *Melipona* and *Scaptotrigona* that lay odor trails [14,18,19]. Multiple recruits will arrive at the food following the odor trail. Although no indications of direction encoding have been found in the agitating runs of stingless bees, the run of *Trigona* is reminiscent of the “jostling runs” of *Apis mellifera* ([1] pp. 278, 307). Esch et al. [15] recorded sound pulses in both *Apis* and stingless bees that varied with distance to the food source. Thus, in both *Apis* and *Trigona,* these runs may stimulate nest mates to search around for food sources, and may give some information about distance. All these examples indicate that possible components of the waggle dance exist in the context of food searching in other insects, and may indicate the existence of pre-adaptations.

Phylogenetic pre-adaptations for the signals in the waggle dance may also be found in a different behavioral context, e.g., warning signals of danger. Bumble bees are famous for their trumpeting sounds [13,20] performed when the colony is attacked or parasitized. Von Frisch [1] compared these warning sounds with the vibrations produced by honeybees during “buzzing dances” and the buzzing tones of *Meliponinae* (p. 315 therein), but we may also see a functional connection to the “stop signals” produced by honeybee dance followers. Stop signals are short, high-frequency signals that occur under rather different conditions. A dance-following bee may produce it to stop a dancer, then engage with her in antennal contacts with her, which then usually leads to food exchange from the dancer to the follower (trophallaxis). Stop signals are also used by nest-scouting bees in their interaction with other dancing-nest scouts [21]. Short pulses of body vibrations are also performed by foraging bees, giving a warning signal to a dancer for a food source that was experienced as dangerous by the stop signal-producing bee [22]. Most likely, there are several different stop signals in *Apis mellifera* that serve different functions in different contexts, and may be differently used in social communication. Not surprisingly, many different terms have been used to characterize these different short pulses (e.g., piping, begging, buzzing, shaking, whooping).

*Apis mellifera* is thought to be a second-order cave-dwelling species [23] deriving from *Apis* ancestors that built their combs freely on branches. The ancestors of *Apis* species belonging to Melliponini and Bombini probably lived in cavities [24]. Transfer of the directional component from the direct guidance by the sun to the gravity field is, therefore, thought to be an evolutionary development associated with cave dwelling. This interpretation is supported by the dance behavior of the Asian dwarf honeybee bee *Apis florea* that dances on the horizontal comb surface under the open sky, pointing the waggle run in the direction of the outbound flight [25]. Von Frisch [1] writes: “In this manner, *Apis florea* confirms our long-cherished suspicion that dance on a horizontal surface with orientation directly according to the sky, represents the more ancient form in which direction was indicated” (p. 304). *Apis mellifera*, when induced to dance under the sky on a horizontal surface, behave similarly. The giant Asian honeybee *Apis dorsata* dances on the vertical comb of its nest, which hangs on a tree branch, under the open sky and uses gravity as the directional guide [26,27]. *Apis dorsata* has not been stimulated to dance on a horizontal surface [25].

Pre-adaptations for the transposition from the visual to the gravity sense are observed in many insect species on the basis of spontaneous switches of walking directions [28,29]. Ants, beetles, cockroaches, and locusts running on a horizontal surface at a spontaneously chosen angle relative to a light source keep the same angle on a tilted surface in the dark. Thus, a transfer between a menotactic running course is kept constant in relation to light and gravity. The rule followed in this behavior is similar to the rule behind directional coding in the dance, upwards in the dark simulates the direction towards the light source (sun).

Summarizing these arguments and taking a cognitive perspective, it is argued that the honeybee dance transmits several messages of increasing complexity: Get ready for feeding, attend the information-carrying animal, perceive and learn physical stimuli associated with the dancing animal, read and learn the symbolically encoded stimuli, create intentions similar to those of the dancer, and act on these intentions.

### 2.2. What Are Intentions and Do Honeybees Have Intentions?

Do insects like many other animals expect future events, predict the value of potential actions, and decide between behavioral options without having access to the indicating stimuli? These cognitive capacities are captured by the term intentionality. Although behavioral analyses document the rich repertoire and the cognitive dimensions of honeybee behavior, intentionality is nearly impossible to prove by behavioral means. Elementary explanation can usually not be rejected, forcing us to apply Occam’s razor and stay with the elementary interpretations. The different approaches in dealing with this epistemological question in the context of dance communication can be characterized by these two contrasting descriptions, written shortly after von Frisch’s discovery:

(1) Haldane and Spurway: “The dance is seen as a highly ritualized intention movement leading to communication which is mainly kinesthetic. The bees which are sent out by the dance fly to the appropriate goal because, by following the dancer, they have automatically carried out the intention movements for the flight. The same principle applies to other communications in which the signal is repeated, including many bird calls” ([7], p. 278).
(2) C.G. Jung et al.: “This kind of message (he means: As encoded in the waggle dance) is no different in principle from the information conveyed by a human being. In the latter case we would certainly regard such behavior as a conscious and intentional act and can hardly imagine how anyone could prove in a court of law that it had taken place unconsciously. Nevertheless it would be possible to suppose that in bees the process is unconscious.” ([30], p. 94).

Haldane and Spurway [7] use the term intention movement in a descriptive way similar to what Darwin 1878 meant by preparatory movement when he wrote about pointing in dogs: “The act of pointing is probably as many have thought the exaggerated pause of an animal preparing to spring on its prey” (cited after [7], p. 266). The preparatory movement or intention movement involves some basic form of automatized or implicit planning of the next movement not necessarily including any expectations about what will happen when the movements are expressed. In contrast, Jung et al. [30] allow for the possibility that the intentional act of dancing includes rich forms of expectations that still can be implicit, not necessarily requiring any form of self-awareness. In neurobiological terminology, intended behavioral acts are based on some sort of neural simulation. Haldane and Spurway [7] and Jung et al. [30] refer to two different kinds of neural simulation, one that is very close to the motor pattern expressed during the outbound flight (statement (1)), or one that includes a multitude of expected perceptual and behavioral conditions that are retrieved from memory and lead to expectations unique for the flight towards the indicated food source (statement (2)). The question then is: Are these neural simulations preparations for the next movement, or are they phenomena of memory retrieval that guide richer forms of expectations? In the case of the waggle dance, the memory of the dancer’s flight is transformed into a ritualized walking pattern that predates, not precedes, the next flight. The following bee performs a close neural simulation that predates and also does not precede its own flight. Unconscious or implicit forms of neural simulations will suffice for both kinds of neural simulation. The difference between the statements of Haldane and Spurway [7] and Jung [30] lies in the level of cognitive processing assumed for neural simulation. The ethological approach expressed in Haldane and Spurway’s wording tries to keep neural simulation as close to motor pattern as possible, leading to a range of problems. For example, the measure of distance has nothing to do with the number of waggles and very little (if at all) with the time or length of the waggle run; the menotactic transformation of the sun azimuth-related waggle run direction does not include any clock dependencies. Bees dance even at night correctly [1], and they dance correctly in relation to elongated landscape features when the sun is not accessible [31]. Follower bees do not perform the same movements as the dancing bee. The cognitive wording of Jung et al. [30] is equally problematic, but it has the advantage that it encourages the design of experiments asking questions that are relevant for the understanding of how an insect brain might work.

I see four characteristics that are essential for a cognitive understanding of intentionality: (1) Identification of the brain as the mental center with its body; (2) the anticipation of future conditions (expectation); (3) the assessment of these conditions with regard to the then-prevailing conditions or needs of one’s own body (evaluation); and (4) the selection of a behavior from two or more options (decision) [32]. These neural operations simulate future conditions, evaluate them, and choose between them as an intrinsic preparatory step for motor expression. Since there is no direct access to intentions by behavioral analyses, it is necessary to search for indirect indications that may allow for the rejection of interpretations not requiring intentions. In the context of the waggle dance, the question centers on the distinction between location as the endpoint of a vector (egocentric reference) and location as defined in spatial relations to other landmarks (allocentric reference). In my view, solving this problem requires evaluating whether recruits make direct flights between dance-indicated locations and other locations they have previously been experienced. It will therefore be important to understand how bees navigate in the explored area around the hive and how they use this knowledge after they have followed a dance. Intentional components of behavior have the potential of activating relevant memories that otherwise might not be accessible. As will be shown here, the memory established during exploratory and foraging flights allows for direct flights between locations, raising the possibility that a cognitive map-like memory may be involved in guiding recruits after following a dance.

### 2.3. Where the Information Comes From: Exploring the Environment

When bees leave the hive for the first time after attending to their indoor duties, they perform orientation flights. In this process, they learn the features of the sun’s compass (when the sun is in which direction), calibrate their distance measurement (odometer) with their eyes, and, above all, learn features of the landscape (prominent landmarks near the hive, the profile of the horizon, distant landmarks) in spatial relation to their hive. They do this quite systematically by covering increasing distances and exploring different directions during consecutive orientation flights [33,34]. After four to six of these orientation flights, they know the area within a radius of about 500 m around the hive as determined by catch-and-release experiments [35]. If the test bees were released in the explored sector, they flew home fast and directed; if, however, they were released in an unexplored sector, they either were lost or took a long time to arrive back at their hive. If bees had already learned the local sun compass conditions and had their odometer calibrated, they needed only one or two orientation flights to locate their hive after the hive was moved to a location outside of the explored area [36]. Foraging flights add information about the landscape as seen in experiments in which foraging bees were released either in the experienced landscape or a novel landscape [37,38]. The foragers returned home successfully when released at any location around the hive within a 500 m distance from the hive but were lost when they were released further away from the hive.

### 2.4. The Memory About the Landscape as a Cognitive Map

After young bees have performed their orientation flights, they follow dances or search on their own for feeding sites. With such experienced bees, we examined the question of which kind of memory they have of the landscape. We trained individual bees to a feeding site, located several hundred meters from the hive. The trained bees traveled the route between the hive and the feeding site many times. In addition to the landscape memory that they formed during the orientation flights, they now also have a special route memory. Our test entailed capturing a bee as it is about to depart from the feeding site after imbibing the sugar solution, and to move it to another location within the explored area (catch-and-release paradigm). If the bee, now fitted with a transponder for flight tracking with a harmonic radar, flew off, it initially behaved as if it had not been displaced. It performed a vector flight, the direction and distance of which would have brought the bee back to the hive had it not been relocated. This is not surprising since the bee was transported to an unexpected location confined in a dark box. Afterwards, it performed search flights, and then, on a straight flight over several hundred meters, it either flew back to the hive directly, or first to the feeding site and then to the hive. Neither the hive nor the feeding site was visible to the bee from the location from which it initiated the straight homing flight. They could not use an odor nor the skyline for guidance. What is amazing is not only that the bee flew back home purposefully but also that it decided whether to do so with a direct flight or with a detour via the feeding site [39]. We concluded from these experiments that bees have a landscape memory that stores many landscape features in their geometrical relationships, like a map. Since this memory is stored in the bee’s brain, we called it a cognitive map. This conclusion has been criticized as there may be more elementary explanations. For example, that, during its orientation flights, the bee could have associated certain landmarks with a direct homing flight [40]. Then, when it passed these landmarks during its search flights, this memory could lead the bee straight home because those landmarks may have been stored together with the direction to the hive as read from the sun compass. Cruse and Wehner [40] also argued that the detour via the feeding site could be similarly explained, whereby the bee would perform a triangulation, calculating from two directions (directly back to the hive and from the hive to the feeding site) a new direction (to the feeding site). If the bee had a cognitive map, it would be independent of the sun compass because it could infer its location and destination from the layout of the landmarks. Acknowledging these considerations, we designed an experiment that allowed us to demonstrate basic properties of a cognitive map.

In bees, as in humans, the internal clock stops when they are anesthetized [41]. This gave us an opportunity to test the role of the sun compass during the homing flight, and to place it in competition with the landmarks [42]. Bees that had been trained to a feeding site as described above were placed under anesthesia for six hours and subsequently released at a different location. The unanesthetized control bees, as expected, first performed their vector flight and then flew back to the hive (some also flew via the feeding site). The anesthetized bees, on the other hand, performed a vector flight that deviated 90 degrees to the east, i.e., they orientated according to the sun azimuth during the vector flight because the sun travels approximately 15 degrees per hour from east to west. If they continued to orientate according to the sun, they would turn accordingly, and because the hive was not located in this direction, it would take them much longer to find their way home. They might even get lost altogether. All this did not happen. After performing the (“wrong”) vector flight, they flew back to the hive just as quickly and purposefully as the control bees. This indicates that they switched from the initially used sun compass course to landmark orientation, as soon as the former proved useless. This experiment demonstrated that bees indeed use a cognitive map.

### 2.5. Integration of Experienced and Communicated Locations in a Common Reference, the Cognitive Map

**Figure 1:**
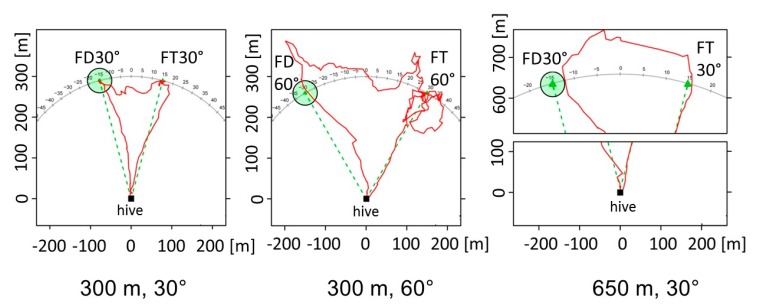
Common memory structure for experienced and dance-indicated location. A group of forager bees was trained to the feeder FT either 300 or 650 m away from the hive. Two or three bees were subsequently trained to FD also 300 or 650 m away from the hive. A few days before the bees danced, FT was closed, and the forager bees followed the dances. When they left the hive, they were equipped with a transponder for harmonic radar tracking. The three figures show representative flight trajectories of recruits that were exposed to three different test conditions (from left to right): 300 m distance, 30° between FT and FD; 300 m distance, 60° between FT and FD; and 650 m distance, 30° between FT and FD (after [43]).

Next, I want to address the question of whether recruited bees relate the waggle dance message to their own navigational memory. Do they apply this information just for their outbound flight performance (using it as a flight instruction), or do they integrate the spatial components of this information into their memory about the landscape? Menzel et al. [43] addressed this question in experiments in which a group of bees foraged at a feeding site (the trained food site, FT) and later experienced that FT did not provide food anymore (Figure 1). As a consequence they gave up foraging at FT and became recruits of two other bees performing dances for a new food site (the dance-indicated food site, FD) at the same distance as FT (two distances were tested: 300 and 650 m) but at an angle of either 30° or 60° to FT (for both distances) as seen from the hive. No odor was involved in training of the foraging group or the dancers. Only two dancers were trained, and their feeder was hidden in the grass. After the feeder was removed, the search flights of bees of the foraging group were not directed to the area to which the dancer bees were trained. Thus, at this stage of the experiments, the bees of the forager group were naive about the location of the feeding place for the dancers. Their landscape memory included only the information from their orientation flights and that of their foraging route. Since these experiments were performed in autumn, the grassland of the test area did not provide natural food sources. This excluded the possibility that the bees from the foraging group had visited the area of the feeder for the dancers before.

We video-recorded the forager group attending the dancers. When one of the dance followers left the dancer and ran to the exit, it was equipped with a radar transponder and its flight recorded. The flights of the recruits from the foraging group depended on their own foraging experience and the information received from dance communication in several respects. (1) The number of outbound flights to either FT or FD depended on the angular difference between FT and FD. (2) The recruits performed a range of novel flight behaviors. In the 30° and 650 m arrangement, some of them deviated from the course toward FD during their outbound flights and crossed over to FT. (3) After arriving at either FD or FT, some of them performed cross flights to the respective other location. These novel shortcuts were seen for both the 30° and 60° arrangement when the distances were 300 m, but only for the 30° arrangement for 650 m distances. It was concluded from these observations that the locations FD and FT are both stored in spatial memory in such a way that bees are able to fly directly from one location to the other following a novel flight route. The decision for FD or FT depended on the number of waggle runs followed by the recruited bee. Following more waggle runs (in our experiment, on average, 15 runs) resulted in FD flights, indicating that the motivation to apply the information collected about FD was enhanced after longer dance following. However, the information about FD had also been learned during shorter dance following, since animals that flew first to FT performed direct flights from FT to FD even after following less than 15 runs. The motivational component appears to remind a recruit about its own foraging experience.

These results not only support the view that learned and dance-communicated locations are embedded in the same spatial memory structure but, more importantly, that bees make decisions with respect to the expected outcome, without having access to the stimuli emanating from the respective locations. These decisions may become apparent right in the moment when the recruits leave the hive or later after they have performed part of their flight to either FT or FD. We thus can conclude that the two options of flying to FT or FD may have already existed in some form of neural activity patterns characteristic for expectations already before leaving the hive and most likely already during dance following.

Dance followers are known to be motivated to fly to their former feeding site after following only a few dance rounds but decide to fly to a new feeding site only after following multiple waggle runs [6]. Our observations corroborate this finding. Thus, dance followers make a decision during dance following in favor of their former experience or against it. Furthermore, bees adjust their food supply from the honey store to the length of the journey which they intend to make [44], indicating a preparation for the expected flight distance. Recruits that had trophallactic interactions with the dancer expect the same quality of the food source (odor, taste) when arriving there [1]. Stop signals performed by following bees are considered as a feedback to the dancer, which requires some form of expectation about the potential outcome if the receiver would apply the dance-indicated behavior [21,45,46,47,48,49]. Stop signals produced are particularly informative in our context when the dancer advertises a food source that is dangerous or of inferior quality [46,49]. The recruit must have identified an experienced location via the symbolic message and related it to the local qualities of that object, which is a form of mapping solely on the basis of symbolic codes. Similar inhibitory signals were found in communication between scouts in a swarm [5]. Overall, these observations clearly document the close connections existing between experienced local properties of the food source (the identity of an object) and its spatial characteristics, as experienced by both the flight route and the symbolic code for it. Object identity and spatial characteristics are thus combined in an object-unique manner. Taken together, the intentions of a recruit during the process of dance following include expectations about the optional travel and its outcome.

### 2.6. What Needs to Be Asked?

Implicit decision making between options may be based on rich information about the potential outcomes or be rather tightly connected to sensory-motor patterns. What kind of intentional processing may be involved in dance communication? Which experiments would inform us about the *intentions* of a dancer and those of a dance follower? As exemplified above, the flight behavior of the follower provides access to what she may expect from the environment if she follows the dance information. The findings presented in Figure 1 give us a hint, but more elementary interpretations like vector addition between self-experienced and dance-communicated vectors cannot be ruled out [40]. I see two areas of research as preparatory steps until it becomes technically possible to monitor high-order brain processes during dance performance, dance following, and/or preparation for the outbound flight.

1. A life history of social interactions and flight activities of individually identified bees may inform us about how dance following and, later in life, dancing depend on experience with the landscape. For example, does dance following of young bees depend on experience collected during their exploratory orientation flights in the sense that only dances are followed that indicate locations within the explored area or in the direction of the explored sector? Are dances not followed if they point into an unexplored landscape sector? In the context of these questions, new experiments should be done to solve the controversy about the famous “lake experiment” [50]. Gould and Gould [50] asked whether recruits of the waggle dance would reject heading towards an “impossible” place (a place in an unexplored area), e.g., a feeding site on a boat in a lake. They reported some evidence in this direction, but their results could not be replicated [51]. However, since the latter authors trained bees with odor at the feeding site, their controls for rejecting guidance by the odor of the feeder on the boat were not fully convincing. Tautz et al. [52] inadvertently found a similar effect as the Goulds when training across a lake in a study on the effect of different landscapes on odometry. In both “lake experiments” of [50,52], the trained bees danced less actively when the station was in the middle of the lake and thus fewer recruits arrived. When the boat reached land again, recruits reappeared but at the point on the shore nearest to the boat, not at the boat itself, indicating an expectation of where the food source could be. This would possibly indicate that the recruits did not choose an impossible location, or the dancer did not dance for an impossible location. However, again, both studies were not controlled well for this effect and no flight trajectories of the trained bees or the recruits were recorded. Tracking bees with a harmonic radar [53] will be required to solve the problem.
2. Given bees’ rich navigational memory, one may ask what exactly is communicated by the waggle dance: Just the outbound vector, or the location of the goal as a spot in the environment defined by its spatial relations to landmarks? Such experiments require uncoupling the vector information from the act of heading to a goal that is not characterized by the endpoint of the vector. This will be possible by releasing the recruits not at the hive entrance, then at different places within the explored area around the hive. If recruits would fly according to the vector information from the dance in the same way as they do when released at the hive entrance, than they apply the flight instruction only. If, however, they also fly towards the real location of the dance vector endpoint, then they refer to additional information that can only derive from their map-like representation learned during exploratory orientation flight. The latter possibility would be particularly strong if one can exclude any foraging of bees in the area around the dance-indicated feeder. An additional experiment could address the question of whether recruits expect certain landscape features on their outbound flights. These expectations can either be any view-based landscape features, spatial relations of objects on the way towards the goal, or particular salient features serving bees as guides toward the goal, like ground landmarks (paths, forest edges, rivers etc.). Again, radar tracking of recruits released at other locations than the hive entrance will be necessary over distances of several hundred meters.

### 2.7. The Honeybee Dance—Not a Language, but a Form of Rich Symbolic Communication

Waggle dance communication is sometimes referred to as language. Von Frisch [1] used this term in a figurative sense, i.e., as a metaphor. However, metaphors can be dangerous if one does not constantly keep in mind that the term has but a figurative meaning. Only human beings have language in the true sense. No animal communicates in a way that includes all the characteristics of human language, the almost unlimited combination of its elements, the link to meaningful structures, and grammar as a set of rules that can give different meanings to the same structures. If we want to understand the level of complexity of the dance communication of bees, we should avoid the term “language”, because it does not apply. This becomes clear when we ask ourselves if dance communication is more comparable to human sign language or the movement ritual in ballet. Certainly, it is not comparable to sign language, because with it we can communicate almost the same contents as with spoken language. What’s more, the same regions of the human brain are responsible for both [54]. Like the waggle dance, dance is a more ballet-like form of communication using a limited number of symbolic elements that are understood as such and that can only be combined in limited ways. Also, ballet does not have semantics or grammar. So, the waggle dance is a symbolic form of communication not a language in the strict sense.

Lindauer [55], following [56,57], referred to six characteristics of language in general that are also found in the bee dance: Broadcasting, semanticity (using symbols), displacement, productivity, interchangeability, and duality. Indeed, the waggle dance broadcasts a message that is interpreted by the receiver and leads to a behavior (productivity). The message is produced independent and at a different place from the location to which it applies (displacement). The symbols used can be applied to different messages, e.g., a feeding place, a new nest site, resin or water source (interchangeability, versatility, duality). However, the symbols used by bees are extremely limited and bound to a rigid code system, and the reference to these characteristics of the human language are mostly only figurative. The approach applied in the literature about the bee dance focuses predominantly on the sender side and mostly ignores the feedback from the receiver and its cognitive domain. This is not surprising because when [55,56,57] discussed these issues they did not know about stop signals (see above), as symbolic feedback from the dance follower to the dancer. Thus, the communication process between the sender and receiver may be much richer than initially thought, and it is so far only through an analysis of the receiver’s behavior that we gain access to the underlying cognitive structure. If such a claim can be substantiated, one has to extend the arguments about the cognitive dimensions of the waggle dance to the dancer since foraging bees frequently change between dancing and dance following. Why should the dancer have less excess to its cognitive processes than the recruit? One way of looking into this question would be to examine more carefully the responses of dancers to stop signals from the followers. Furthermore, it will be important to understand better how a dancer selects locations to indicate after she has experienced more than one feeder. For example, do dancers that had experience exclusively with nectar and exclusively pollen sources at different locations advertise for one or the other food in response to changes in the need of the colony for nectar or pollen? Questions like these require a full life history of experience of individual bees both inside and outside of the colony. This is not an impossible task give the power of video recordings and radar tracking.

## 3. Conclusions

I propose a cognitive view of waggle dance communication. The dancing bee may perform as if she is flying to a selected location, activating neural substrates characteristic of the perceived and/or expected experience during flight, and the follower bee reads this message by incorporating it into its navigational memory. The relevant memories may become activated specifically for compass-related measures, object identities (landmarks, views), salient landscape features, and their spatial relations as stored during exploratory and foraging flights. These memories are implicit, but they are sorted according to their cognitive domains, describing the resources in terms of when, what, where, and how to get there. As long as we lack any neurophysiological approach to explore the underlying brain mechanisms, behavioral analyses will be more informative when focusing on the receiver of the dance message, the following bee, since her subsequent behavior can be more easily observed and manipulated. The data collected in these attempts need to match the natural dimensions of flight behavior, so far only possible with harmonic radar tracking. Feedback signals from the dance follower to the dancer are equally informative because they indicate communication on a symbolic level that by definition requires reference to bees’ memories.
